# The General Medical Council

**Published:** 1898-12-03

**Authors:** 


### THE GENERAL MEDICAL COUNCIL.

The sixty-sixth session of the General Medical Council was opened on Tuesday, November 22nd, by an address from Sir William Turner, the President, in which he gave the customary brief summary of action which had been taken during the recess, and of the business which would have to be considered. The Council received and acknowledged the usual tabular information with reference to the examinations for admission into the medical service of the army and of the Government of India, the tables of the same kind with reference to the Royal Navy not being complete in time for presentation on this occasion. Communications on the subject of the employment of unqualified assistants were referred to a committee for report during the session; and certain documents were ordered to be placed at the disposal of members of the Council for purposes of reference, and to be arranged in a room provided for the occasion. The Council rose at four o'clock, in order to permit the meeting of several of its committees.

On Wednesday, the 23rd, the first business was the consideration of a charge of covering brought against Mr. C. A. Duckett; and it differed from ordinary cases of the same kind in the fact that Mr. Duckett was not the proprietor of the practice in which the alleged covering took place, but was a temporary substitute during the absence of the proprietor. After a careful hearing, it was decided that the charge had not been proved to the satisfaction of the Council. The next business was the consideration, *in camerâ*, of certain recommendations from the Executive Committee as to applications, from persons whose names had been erased from the Register for various offences, for restoration thereto. The applications were refused in the cases of William John Patterson, Robert Ingram Robertson, Robert Alexander Douglas Lithgow, and Herbert Tibbits; and the consideration of the application of Alfred Freeman was adjourned, with a reference of documents in the case to the Royal College of Physicians of Edinburgh. Other notices of motion for the inspection of documents were accepted as urgent, and the motions were agreed to.

On Thursday, the 24th, the Council considered a charge of covering an unqualified assistant made against Mr. Frederic Mercer, and decided that it had not been proved to their satisfaction. The name of John Lloyd Whitmarsh was erased from the Medical Register on certificate from the Central Criminal Court that he had been convicted of felony; and a charge against Frederic Alfred Fisher of issuing grossly discreditable advertisements was considered. The Council adjudged that Frederic Alfred Fisher had been guilty of infamous conduct in a professional respect, and directed the erasure of his name from the Register. The remaining business of the day was conducted *in camerâ*.

On Friday, the 25th, the whole of the day was taken up in hearing a charge of covering, brought by Dr. John Jones, of Clydach, against Mr. James Havard Jones of the same place. On Saturday the Council deliberated *in camerâ*, and decided that the charge had not been proved to their satisfaction. A discussion on a motion for a transference of the duties now discharged by the Penal Cases Committee to the three Branch Councils was adjourned; and certain motions for alterations in the standing orders were deferred until after this point had been decided.

On Monday, the 28th, the Council rejected a motion by Mr. Horsley to alter the arranged order of business in favour of a discussion upon a case pending before the Queen's Bench Division of the High Court, and proceeded to discuss a report from the Executive Committee with reference to some proposed alterations in the standing orders as affecting penal cases. Certain changes were made, and the orders, as altered or amended, were referred to the legal advisers of the Council for their consideration. Mr. Brown had given notice of three motions, the first to report to the Privy Council that an additional direct representative for England was desirable, and the other two to assert the same thing with regard to Scotland and Ireland. He was granted leave to withdraw the last two from the programme. A report from the Education Committee with regard to preliminary examinations in general knowledge was brought up by the chairman of the committee, Sir John Batty Tuke, and was discussed until the hour of adjournment.

On Tuesday, the 29th, the Council completed its discussion of the above-mentioned report, which was adopted; and the committee was asked to inform the Council, at the May meeting, with regard to the date at which a demand for a higher standard of preliminary examination might reasonably be brought into operation. Reports were received from Dr. Coupland, the inspector appointed by the Council to report upon the sufficiency of the qualifying examinations conducted by the Apothecaries' Hall of Dublin, with the aid of assistant examiners in surgery appointed by the Council. The examinations were described as "sufficient," but a tendency to over-marking was made a subject of comment. The report of a committee appointed to confer with the promoters of a Bill for the regulation of midwives was considered, and a committee was again appointed to keep the matter under observation. A report was received from a committee appointed to approach the Government with reference to the Companies Acts Amendment Bill, and for the purpose of obtaining the insertion of provisions to render illegal the conduct of medical, surgical, or dental practice by joint-stock companies. Reports from the Public Health Committee, on different interpretations placed upon the Council's regulations with regard to diplomas in public health, and from the Students' Registration Committee were read and considered, and the Council adjourned.

